# The Application of Proportional Universalism to Early Childhood Intervention in Japan: A Cross‐Sectional Survey

**DOI:** 10.1002/puh2.70188

**Published:** 2026-01-19

**Authors:** Kimiko Ueda

**Affiliations:** ^1^ Faculty of Health and Well‐Being Kansai University Osaka Japan

**Keywords:** child and family centers, early childhood intervention (ECI), population approach, proportionate universalism, targeted approach

## Abstract

The application of proportionate universalism has been proposed as an approach that may promote timely access to necessary early childhood intervention (ECI) for children with disabilities. Child and family (CF) centers have recently been established in Japan through the merging of maternal and children's comprehensive support (MCCS) centers using a population approach and comprehensive child and family support centers using a targeted approach. However, reports on how proportionate universalism can be implemented in practice are lacking. Therefore, the present study aims to examine Japan's challenges and actionable recommendations in new CF centers. An anonymous, online survey was conducted on mothers of children aged ≤3 years who had been randomly selected from a special panel throughout Japan. The survey included items on the characteristics of the participants, whether they used MCCS centers and were concerned about their child's development, and whether they were receiving any follow‐up. Responses were obtained from 866 mothers. Among mothers who knew about MCCS centers, those with children with developmental delays who received follow‐up care were significantly more likely to use them (*p* = 0.003). Overall, 75.4% of the children with developmental concerns were connected to not only hospital visits but also ECI by government agencies; these families were also significantly more likely to use MCCS centers (*p* = 0.004). These findings suggest that when children's developmental delays are noticed, ongoing involvement at an MCCS center is associated with timely and appropriate referrals. The services to be provided at CF centers should be based on proportionate universalism.

## 1 Introduction

Parenting support is particularly important for cases involving children with developmental delays [1, 2, 3]. Such developmental delays may lead to parenting problems, as well as problems for the parents themselves [4, 5, 6]. When parents suspect and have concerns that their child may have a developmental delay, public health nurses and other health professionals can help them access early childhood intervention (ECI) if needed.

An ideal health system should enable parents with concerns about their child's developmental delay to access ECI promptly [7, 8]. Vulnerable groups, such as parents with children with disabilities, single parents, and parents with mental illnesses, have always existed in the field of perinatal, maternal, and child health. Proportionate universalism, which integrates a targeted approach for the vulnerable using a population approach, helps ensure that such vulnerable groups have access to the services they need for equitable health outcomes [9, 10, 11, 12]. Proportionate universalism means that services are offered to all families, but the amount and type of support increase for those who need more help [13]. For example, all parents receive routine maternal and child health services, whereas those with additional needs receive enhanced follow‐up and individualized support. This approach emphasizes a cross‐sector collaborative services delivery model, which can facilitate access to early intervention for children and their families, regardless of their level of vulnerability [13, 14].

There is growing concern about increasing health disparities in Japan, and health problems are accumulating for the vulnerable [15]. Society at large believes that parenting support is extremely important for preventing and solving child poverty and correcting disparities. The Japanese government sustains an ongoing care system for all pregnant women, children, and their families while providing targeted support through financial and professional assistance, taking social and health disparities into account. In addition, regarding services for children, such as health checkups and the individual distribution of maternal and child health handbooks, public health workers provide consultations and identify children and families who face social and health challenges and provide support through telephone counseling and home visits. On the other hand, delays in providing necessary support remain a major challenge because of a lack of coordination among facilities. To address this issue, the following measures aim to ensure that proportionate universalism functions effectively across perinatal, maternal, and child health‐care support, especially in ECI.

The Japanese government established maternal and children's comprehensive support (MCCS) centers nationwide by the end of 2020 with the expectation that such centers would be effective for promoting child and family health. These MCCS centers, which use a population approach [16] in which all pregnant, parturient, and postpartum women and young children are targeted regardless of the presence of any risks related to pregnancy, birth, or parenting, were to serve as municipal bases capable of providing one‐stop support services related to pregnancy, birth, and parenting.

For children requiring more specialized support, such as those with disabilities and/or diseases, MCCS centers adopt a targeted approach [17] by collaborating with municipal comprehensive child and family support (CCFS) centers. CCFS centers, which were established in April 2017, are responsible for supporting children with disabilities. The early identification of delays and the initiation of appropriate ECI are important for both children with developmental delays and their parents. However, the transition from MCCS to CCFS centers has not been smooth, which has led to services sometimes being delayed.

New child and family (CF) centers were established in April 2024 by integrating MCCS centers with CCFS centers. The main purpose of establishing these integrated CF centers was to strengthen further the consultation services in terms of both health and welfare. However, reports on how proportionate universalism can be implemented in practice are lacking [18]. Therefore, the present study aimed to examine Japan's challenges and actionable recommendations in regard to CF centers as practical examples of proportionate universalism by conducting a preliminary survey of MCCS centers in the area of ECI.

## 2 Methods

In April 2022, before the opening of CF centers through the integration of MCCS and CCFS centers, 96% and 37% of the municipalities in Japan had established MCCS and CCFS centers, respectively. Clarifying the status of MCCS center utilization and referrals to ECI could help guide actionable recommendations for newly established CF centers. Therefore, a survey of MCCS awareness and utilization in 2022 was conducted as a preliminary study.

An anonymous, online, self‐administered survey was conducted for 1 month starting in February 2022 on mothers of children aged ≤3 years who had been randomly selected from a special panel of approximately 330,000 samples throughout Japan (Cross Marketing Inc., Tokyo, Japan). The distribution of the online questionnaire was terminated when the number of responses for each age group and sex reached approximately 100.

This study was approved by the Ethics Committee of the author's institution. The survey included items on the characteristics of the participants (e.g., age, place of residence, marital status, family members living together, educational level, and employment status), whether they used MCCS centers and were concerned about their child's development, and whether they were receiving any follow‐up, including ECI, in regard to their child's development.

The *χ* ^2^ test was used to determine whether there were any statistically significant differences in MCCS center usage and characteristics between mothers who were and were not receiving follow‐up care. The same test was performed regardless of whether there was a difference in places that recommended follow‐up between those who used MCCS centers and those who did not.

## 3 Results

Responses were obtained from 866 mothers (mean age, 34 years). The characteristics of the mothers are shown in Table 1. In total, 31.8% of the mothers reported having used MCCS centers, 36.6% had concerns about their child's development, and 57.7% were not receiving follow‐up care. In cases where child development was a concern, children with unmarried or divorced mothers were significantly more likely to be followed (*χ* ^2^ (4) = 12.681, *p* = 0.013). On the other hand, among mothers who knew about MCCS centers, those with children with developmental delays who received follow‐up care were significantly more likely to use them (*χ* ^2^ (1) = 8.609, *p* = 0.003) (Tables 1 and 2). Mothers with a lower educational background who knew about MCCS centers were significantly more likely not to be receiving follow‐up care (*χ* ^2^ (1) = 4.213, *p* = 0.04) (Table 2). Among mothers whose children were being followed, the institutions that indicated a need for children's developmental follow‐up and were currently providing developmental follow‐up are shown in Table 3. In total, 60.4% of those using MCCS centers were recommended by governmental institutions. Among mothers whose children were being followed, 39.6% were seeking follow‐up without any indications from any sources. In addition, 75.4% of the children with developmental concerns were connected to not only hospital visits but also ECI by government agencies; these families were also significantly more likely to use MCCS centers (*χ* ^2^ (1) = 8.136, *p* = 0.004).

**TABLE 1:** Characteristics of mothers of children aged ≤3 years according to the child's developmental status.

		Child's developmental status			
	Total	No concerns (*n* = 549)	Concerns without follow‐up (*n* = 183)	Receiving follow‐up (*n* = 134)	*p*
Mean age (years)	34.3	34.2	34.4	34.6	0.222
Marital status					
Unmarried	11 (1.3%)	2 (0.4%)	4 (2.2%)	5 (3.7%)	
Divorced/Bereaved	37 (4.3%)	21 (3.8%)	8 (4.4%)	8 (6.0%)	
Married	818 (94.5%)	526 (95.8%)	171 (93.4%)	121 (90.3%)	0.013
Employed					
Yes	470 (54.3%)	305 (55.6%)	89 (48.6%)	74 (56.1%)	
No	396 (45.7%)	244 (44.4%)	94 (51.4%)	58 (43.9%)	0.861
Habitation area; population of municipality					
≥500,000	305 (35.3%)	191 (34.8%)	63 (34.4%)	51 (38.1%)	
200,000 to <500,000	177 (20.4%)	114 (20.8%)	40 (21.9%)	23 (17.2%)	
<200,000	384 (44.3%)	244 (44.4%)	80 (43.7%)	60 (44.8%)	0.861
More than one child					
Yes	273 (31.5%)	182 (33.2%)	49 (26.8%)	42 (31.3%)	
No	593 (68.5%)	367 (66.8%)	134 (73.2%)	92 (68.7%)	0.274
Living with grandmother and/or grandfather					
Yes	802 (92.6%)	513 (93.4%)	167 (91.3%)	122 (91.0%)	
No	64 (7.4%)	36 (6.6%)	16 (8.7%)	12 (9.0%)	0.466
Educational background					
Junior/High school	204 (23.6%)	126 (23.0%)	51 (27.9%)	27 (20.1%)	
Junior college/university/graduate school	662 (76.4%)	423 (77.0%)	132 (72.1%)	107 (79.9%)	0.239

*Note:* *p* values were calculated using chi‐square tests.

**TABLE 2:** Developmental follow‐up, maternal and children's comprehensive support (MCCS) centers use, and maternal education among mothers aware of MCCS centers.

	No follow‐up (*n* = 135)	Follow‐up (*n* = 79)	*p*
Used an MCCS center			
Yes (*n* = 102)	54 (40.0%)	48 (60.8%)	0.003
No (*n* = 112)	81 (60.0%)	31 (39.2%)	
Maternal educational			
Junior/High school (*n* = 49)	37 (27.4%)	12 (15.2%)	
Junior college/university/graduate school (*n* = 165)	98 (72.6%)	67 (84.8%)	0.04

*Note:* *p* values were calculated using chi‐square tests.

**TABLE 3:** Institutions identifying and providing developmental follow‐up for children under follow‐up.

	Using MCCS centers (*n* = 48)	Not using MCCS centers (*n* = 86)	*p*
Institutions identifying the need for follow‐up			
Governmental institutions (ECI), including MCCS and health centers (*n* = 64)	29 (60.4%)	35 (40.7%)	0.088
Medical institutions (*n* = 17)	5 (10.4%)	12 (14.0%)	
Mother or family judgment (*n* = 53)	14 (29.2%)	39 (45.3%)	
Institutions providing developmental follow‐up			
Medical institutions and governmental institutions, including MCCS and health centers (*n* = 101)	43 (89.6%)	58 (67.4%)	0.004
Medical institutions only (*n* = 33)	5 (10.4%)	28 (32.6%)	

*Note:* *p* values were calculated using chi‐square tests.

Abbreviations: ECI, early childhood intervention; MCCS, maternal and children's comprehensive support.

## 4 Discussion

The results of the present study suggest that when children's developmental delays are noticed, ongoing involvement at an MCCS center is associated with timely and appropriate referrals. Such children received follow‐up care for their developmental delays mainly at governmental child development support centers and medical institutions. In addition, the use of MCCS centers was associated with receiving more developmental follow‐up at governmental institutions, including MCCS and health centers. In Japan, governmental institutions provide ECI, which means that children with disabilities receive more specialized support for ECI than follow‐up in hospitals alone. In this study, lower maternal educational background was associated with a lower likelihood of receiving developmental follow‐up, even among mothers who were concerned about their child's development. As CF centers have only been used since 2024, in other words, when proportionate universalism is in full swing, it is necessary to take socioeconomic factors such as the mother's level of education into account. In addition, the strategic awareness and promotion of the use of CF centers are clearly important. For ECI to be effective, it is necessary to have a system that enables parents to consult with health service providers easily when they have concerns about their child's development.

Whether integrated services have a positive impact on actual child and family outcomes remains a topic of debate [19]. However, intensive continuity care by professionals has been shown to have a positive impact on family mental health [20]. Even if parents have concerns about developmental delays in their child, it does not mean that the child necessarily has a developmental delay, and it is possible that the parents need support in the form of reassurance. They also require specialized health‐care or welfare providers, such as CF centers, that they can easily consult with regarding their concerns about their child's growth and development.

At new CF centers, specialized services for developmental delays and support from public health nurses are accessible at the same place, which enables children and their families to receive continuous support. In addition, the increased numbers of staff allow for more efficient operations, as time‐consuming exchanges of information on the same case can be eliminated. CF centers integrating MCCS centers, which use a population approach, and CCFS centers, which use a targeted approach, are therefore expected to provide improved ECI for children with disabilities through the implementation of proportionate universalism. To achieve this, it is critically important to instill awareness that CF centers are welcoming and accessible institutions in the community.

The present study has several limitations. First, as the questionnaire was anonymous and internet‐based, it may have involved a selection bias. Respondents were likely to be mothers who were relatively familiar with internet use and had sufficient time to respond to an online survey. In contrast, mothers who were concerned about their child's developmental delays are more intensively engaged in childcare, ECI, or hospital visits, and therefore may have been less likely to spend time on responding to internet surveys. As a result, mothers who require support but lack sufficient time or psychological capacity may be underrepresented.

Second, although the study identified associations between the use of MCCS centers, maternal educational background, and developmental follow‐up, alternative explanations should be considered. For example, mothers with higher educational backgrounds may have greater access to information [21], stronger health literacy [22], or more proactive help‐seeking behaviors [23], which could independently influence both MCCS utilization and follow‐up care, regardless of MCCS center use itself. Similarly, regional differences in service availability or local referral practices may have influenced patterns of follow‐up.

Third, the use of MCCS centers among mothers may vary according to the specific services provided by each center; however, the present study did not examine the content, intensity, or quality of services provided at individual centers. Therefore, differences between centers could not be examined, and the observed associations should be interpreted cautiously. In addition, given the cross‐sectional design, causal relationships between MCCS center use and developmental follow‐up cannot be inferred.

To investigate the actual support provided at such centers and the reasons for using them, and to clarify the detailed reasons of those who do not use them, additional research is needed with larger and more diverse samples, longitudinal designs, and detailed assessments of center‐level services.

## 5 Conclusions

The application of proportionate universalism to perinatal, maternal, and child health services is expected to be effective in helping to identify children with disabilities and granting them access to ECI in a timely manner. In April 2024, CF centers were established in Japan by merging MCCS centers, which use a population approach, with CCFS centers, which use a targeted approach. Of note, MCCS and CCFS centers should not just simply be integrated; rather, their functions need to be fully coordinated. Consequently, CF centers could provide much‐needed support for children with disabilities through an ideal proportionate universalism approach. The results of the present questionnaire survey indicated that mothers of children with disabilities who could access ECI were more likely to use MCCS centers. This finding suggests that awareness of the services provided by CF centers should be promoted throughout the community and that the services should be readily available to all expectant mothers, children, and their families.

## Author Contributions

Conceptualization, Data curation, Formal analysis, Funding acquisition, Investigation, Methodology, Project administration, Resources, Software, Validation, Visualization, Writing.

## Funding

This study was supported in part by grants from the Japan Society for the Promotion of Science (JSPS), KAKENHI Grant Numbers 19K02638 and 22K02394.

## Conflicts of Interest

The author declares no conflicts of interest.

## Data Availability

Data are available on request from the author.
